# A Family of Optimal Linear Functional-Repair Regenerating Storage Codes

**DOI:** 10.3390/e27040376

**Published:** 2025-04-01

**Authors:** Henk D. L. Hollmann

**Affiliations:** Institute of Computer Science, University of Tartu, Tartu 50409, Estonia; henk.hollmann@ut.ee

**Keywords:** distributed storage, storage codes, functional repair, cut-set bound, MDS codes

## Abstract

We construct a family of linear optimal functional-repair regenerating storage codes with parameters {m,(n,k),(r,α,β)}={(2r−α+1)α/2,(r+1,r),(r,α,1)} for any integers r,α with 1≤α≤r, over any field when α∈{1,r−1,r}, and over any finite field $F_{q}$ with q≥r−1 otherwise. These storage codes are Minimum-Storage Regenerating (MSR) when α=1, Minimum-Bandwidth Regenerating (MBR) when α=r, and represents extremal points of the (convex) attainable cut-set region different from the MSR and MBR points in all other cases. It is known that when 2≤α≤r−1, these parameters cannot be realized by exact-repair storage codes. Each of these codes come with an explicit and relatively simple repair method, and repair can even be realized as help-by-transfer (HBT) if desired. The coding states of codes from this family can be described geometrically as configurations of r+1 subspaces of dimension α in an *m*-dimensional vector space with restricted sub-span dimensions. A few “small” codes with these parameters are known: one for (r,α)=(3,2) dating from 2013 and one for (r,α)=(4,3) dating from 2024. Apart from these, our codes are the first examples of explicit, relatively simple, optimal functional-repair storage codes over a small finite field, with an explicit repair method and with parameters representing an extremal point of the attainable cut-set region distinct from the MSR and MBR points.

**Data Set License:** CC-BY

## 1. Introduction

The amount of data in need of storage continues to grow at an astonishing rate. The International Data Corporation (IDC) predicts that the Global Datasphere (the total amount of data created, captured, copied, and consumed globally) will grow from 149 zettabytes in 2024 [1], to 181 zettabytes by the end of 2025 [2,3], and to an estimated 394 zettabytes in 2028 [4] (a zettabyte equals $10^{21}$ bytes). These developments may even be accelerated by the advancement of generative AI models. In view of these developments, the importance of efficient data storage management can hardly be underestimated. A major challenge is to devise storage technologies that are capable of handling these huge amounts of data in an efficient, reliable, and economically feasible way.

### 1.1. Distributed Storage Systems and Storage Codes

In modern storage systems, data storage is handled by a *Distributed Storage System* (DSS). A DSS stores data across potentially unreliable storage units commonly referred to as *storage nodes*, which are typically located in servers in data centers in widely different locations. Efficient update and repair mechanisms are critical for maintaining stability, especially during node failures [5]. To handle the occasional loss of a storage node, the DSS employs *redundancy*, in the form of a *storage code* [6,7]. Often, a DSS simply employs *replication*, where the storage code takes the form of a *repetition code*. But nowadays, many storage systems such as Amazon S3 [8]; Goole File System [9] and its successor Colossus [10]; Microsoft’s Azure [11,12,13]; and Facebook’s storage systems [14,15], offer a storage mode involving a (non-trivial) erasure code. Especially for *cold data* (data that remains unchanged, for example for archiving), but also for warm data (data that needs to be updated only occasionally), non-trivial erasure codes such as Reed–Solomon (RS) codes, Locally Repairable Codes (LRCs) or Regenerating Codes (RGCs) are considered or already applied [7,16]. For example, Microsoft Azure employs a Reed–Solomon code for archiving purposes [11]. Hadoop implements various Reed–Solomon (RS) codes [17,18], and the implementation of other codes such as HTEC has been proposed, see, e.g., [19]. The Redundant Array of Independent Disks (RAID) standard RAID-6 specifies the use of two-parity erasure codes, see, e.g., [20]. Huawei OceanStor Dorado [21,22] employs Elastic EC, offering choice between replication and EC, for example RAID-TP (triple parity), and IBM Ceph also offers a choice of EC profiles [23,24] (see also [25]). Several good overviews of modern storage codes and their performance are available, see for example [16,26,27,28,29]. For a general and recent reference on storage systems, see [30], and for an overview of Big–Data management, see [31].

### 1.2. Node Repair

In the case of a lost node, the DSS uses the storage code to repair the damage. During repair, the DSS introduces a *replacement node* (sometimes called a *newcomer* node) into the system and downloads a small amount of data from some of the remaining nodes, referred to as the *helper* nodes; the data obtained is then used to compute a block of replacement data that is to be placed on the replacement node. This process, commonly referred to as *node repair*, comes in two variations. In the simplest repair mode, referred to as *exact repair* (ER) [32,33], the data stored on the newcomer node is an *exact* copy of the data stored on the lost node. A more subtle repair mode, first considered in [6], is *functional repair* (FR), where the replacement data need not be an exact copy of the lost data, but is designed to maintain the possibility of recovering the data that was originally stored, as well as to maintain the possibility for future repairs. An ER storage code can be thought of as an *erasure code* that enables efficient repair. In contrast, an FR storage code can be seen as a *family* of codes, all having the same parameters, where an erasure in a codeword from a code in the family is corrected into a codeword from possibly another code in the family [29] (Section 3.1.1). We define and discuss *linear* FR storage codes in detail in Section 3, and describe an example in Example 1. For a formal definition of general FR storage codes, we refer to [29] (Section 3.1.1).

### 1.3. Effectiveness of a Storage Code

Key considerations for measuring the effectiveness of a storage code are the *storage overhead* and the *efficiency of the repair process*. The storage overhead is determined by the fraction of *redundancy* employed by the code, and is measured by the *rate* of the code. Efficient repair, first of all, requires an easily implementable repair algorithm. Other important factors are the amount of data that needs to be transferred during repair, referred to as the *repair bandwidth*, and the amount of *disk I/O*, the number of times that a symbol is accessed on disk. In addition, it is desirable to limit the number of nodes that participate in the repair process, known as the *repair degree* [6] or *repair locality* [34,35].

In general, the data that is transferred by a helper node during repair may be *computed* from the available data symbols stored in that node. If each of the helper nodes simply transfers a *subset* of the symbols stored in that node, then we speak of *help by transfer (HBT)* [26,29]; if, in addition, no computations are done either at the newcomer node then we speak of *repair by transfer (RPT)* [36,37]. We say that a storage code is an *optimal-access* code if the number of symbols read at a helper node equals the number of symbols transferred by that node [26,29,38].

### 1.4. Regenerating Codes and Locally Repairable Codes

Research into storage codes has diverged into two main directions. Regenerating codes (RGCs) investigate the possible trade-off between the storage capacity per node and the repair bandwidth (the total amount of data download during repair), which is determined by the cut-set bound [6]. On the other hand, Locally Repairable Codes (LRCs) study the influence of the *repair degree*, the number of helper nodes that may be contacted during node repair [34,35,39]. A good overview of the different lines of research on codes for distributed storage and the obtained results can be found in [40].

We first discuss an often-used model for storage codes, see, i.e., [6,26,27,29]. A *regenerating code* (RGC) with parameters ${\left\{m,\left(n,k\right),\left(r,\alpha ,\beta \right)\right\}}_{q}$ is a code that allows for the storage of *m* information symbols from some finite field $F_{q}$, in encoded form, onto *n* storage nodes, each of which being capable of holding α data symbols from $F_{q}$. We will refer to α as the *storage capacity* or the *subpacketization* of a storage node. The parameter *k* indicates that at all times, the original stored information can be recovered from the data stored on *any* set of *k* nodes. It is assumed that *k* is the smallest integer with this property; since any set of *r* nodes can repair all the remaining nodes, we then have k≤r. Note that the *rate* of the code is the fraction m/(nα) of information per stored symbol. The resilience of the code is described in terms of a parameter *r*, referred to as the *repair degree*, and a parameter β, referred to as as the *transport capacity* of the code. If a node fails, then a *replacement node* is introduced into the system, which is then allowed to contact an *arbitrary* subset of size *r* of the remaining nodes, referred to as the set of *helper nodes*. Each of the helper nodes is allowed to compute β data symbols, which are then sent to the new node, which uses this data to compute a *replacement block*, again of size α. Therefore, the repair bandwidth γ of a RGC satisfies γ=rβ. It has been shown [6] that the parameters of an RGC satisfy the *cut-set bound*

(1) $$m\leq \sum_{i=0}^{k-1}\min\left(\alpha ,\left(r-i\right)\beta \right).$$

**Figure 1:**
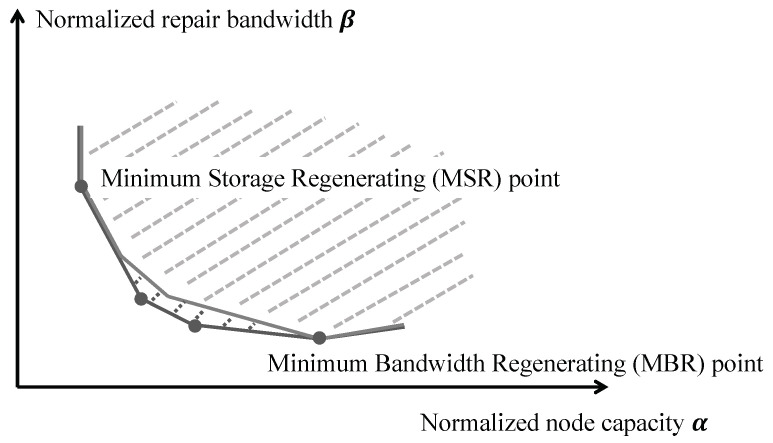
The typical achievable region for functional repair and for exact repair when k=4, with fixed *m* and *r*.

Remarkably, the cut-set bound is independent of *n* (but *n* does influence the required field size *q* for code construction). For fixed *m*, *k*, and *r*, the equality case in (1) takes the form of a piece-wise linear curve that represents the possible trade-off between the storage capacity α and the transport capacity β. Note that we have α≥m/k (since *k* nodes can recover the data) and β≥α/r (since *r* nodes can repair); the points on the curve where α=m/k with minimal β (so with β=α/(r−k+1)) and β=α/r with minimal α (so with $\alpha =rm/(rk-\left(k^{2}-k\right)/2))$ are referred to as the Minimum Storage Regenerating (MSR) and Minimum Bandwidth Regenerating (MBR) points, respectively. It is easily verified that the achievable region determined by (1) is convex and has precisely *k* extreme points (also referred to as *corner points*), see Figure 1. We review the cut-set bound in detail in Section 4.

An *optimal* RGC is an RGC with parameters that attain the cut-set bound (1). It has been shown [41] (Theorem 7) that the MSR and MBR points are the only corner points that can be achieved by exact-repair RGCs; indeed, the only points on the cut-set bound between the MSR and MBR points that can be achieved by ER RGCs are the MSR and MSB points, with the possible addition of a small line segment starting at the MSR point and not including the next corner point. In fact, it is conjectured that the achievable region for ER RGCs is described by the (identical) parameter sets of Cascade codes [42] and Moulin codes [43]. Conversely, it has been shown [44] that every point on the cut-set bound is achievable by functional-repair RGCs; however, these codes are not (or not really) explicit, require a very large field size, and do not come with a repair algorithm. As far as we know, the only known explicit optimal FR RGCs are the partial exact-repair MSR codes with m=2k from [37], the explicit k=n−2 HBT “FMSR” codes in [45] (see also the “random” NCCloud HBT codes in [46] and the non-explicit k=2 MSR codes in [47]), and the two explicit optimal FR RGCs from [48] and from [49,50]. Therefore, it is of great interest to construct “simple” FR RGCs with a small field size, in corner points different from the MSR and MBR points.

A *Locally Repairable Code (LRC)* also has parameters ${\left\{m,\left(n,k\right),\left(r,\alpha ,\beta \right)\right\}}_{q}$, where *m*, *n*, *k*, α, and β have the same meaning as for RGCs, but now we just require that repair of a failed node is always possible if we employ a *specific* set of *r* helper nodes (i.e., we are allowed to *choose* the *r* helpers). In [51,52] the maximal *rate* of such codes (without any constraint on *k*) was investigated, and in [52], it was conjectured that for the case where r+1∣n, the optimal rate is achieved by partitioning the *n* storage nodes into *repair groups* of size r+1 and, within each repair group, using an ${\left\{m,\left(n,r\right),\left(r,\alpha ,\beta \right)\right\}}_{q}$*optimal* RGC, so with *m* attaining equality in (1). This partly explains our interest in RGCs with these parameters in this paper. It is an interesting problem to investigate optimal codes for the case where r+1 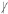 *n*.

### 1.5. Our Contribution

Many existing storage codes employ MDS codes or, essentially, *arcs* in projective geometry, in their construction. Some examples are the MBR exact-repair codes obtained by the matrix-product code construction in [53], the MSR functional-repair codes in [37] and in [47], and the exact-repair Moulin codes in [43]. In this paper, we use MDS codes to construct *explicit* optimal linear RGCs with n−1=r=k, β=1, and with α an integer with 1≤α≤r, so with m=(2r−α+1)α/2, which we refer to as *(r,α)-regular codes*. In fact, we show that the existence of (r,α)-regular storage codes is *equivalent* to the existence of an ${\left[r,\alpha ,r-\alpha +1\right]}_{q}$ MDS code, so they can be realized over finite fields $F_{q}$ with q≥r−1, and even as binary codes if r−α≤1. These codes come with a relatively simple repair method, and we show that, if desired, they allow for help-by-transfer (HBT) repair. The parameters of these codes achieve the *r* extremal points of the achievable cut-set region for varying α. Note that by employing the obvious *space-sharing technique* [37], we can use the two storage codes in consecutive extremal points on the cut-set bound (1) to also achieve the points between these extremal points. Our construction is based on what we call (r,α)-regular configurations, collections of r+1 subspaces of dimension α in an ambient space of dimension *m* with restricted sub-span dimensions (such configurations where called (r,α−1)-good in [48] and [49], see also [51] (Example 3.3)).

The contents of this paper are organized as follows. In Section 2, we introduce some notation and we recall various notions from coding theory, and in Section 3, we review linear storage codes. We revisit the cut-set bound in Section 4, where we also show that in optimal RGCs with k>1, no two nodes store identical information; in addition, we show that if *s* an integer such that (s−1)β≤α≤sβ, then any r−s+1 nodes carry *independent* information, that is, together they carry an amount of information equal to (r−s+1)α. In addition, in the case where r=k, we derive an inequality that motivates our definition of (k,r,s,β)-regular configurations in Section 5, where we also construct such configurations for all relevant parameters. The (k,r,s,β)-regular configurations with k=r, β=1, and α=s are called *(r,α)-regular*. In Section 6, we investigate the structure of such configurations. Section 7 contains our main results. Here, we show that the repair of a lost node in an (r,α)-regular coding state necessarily involves an MDS code, thus providing a lower bound for the size of the finite field for which an (r,α)-regular storage code can be constructed. Theorems 3 and 4 together demonstrate existence of (r,α)-regular codes for all feasible pairs (r,α), and include precise and simple repair instructions for the corresponding codes. In Section 8, we describe how to obtain smaller (r,α)-regular storage codes with extra symmetry, involving only (r,α)-regular configurations of a more restricted type. Finally, in Section 9, we present some conclusions.

## 2. Notation and Preliminaries

For a positive integer *n*, we define [n]:={1,…,n}. We write $F_{q}$ to denote the (unique) finite field of size *q*. For two vectors $a=(a_{1},\ldots ,a_{m})$ and $b=(b_{1},\ldots ,b_{m})$ in some vector space $V≅F_{q}^{m}$, and for a k×m matrix $M=(M_{i,j})$ with entries in $F_{q}$, define the *dot product* $a\cdot b:=a_{1}b_{1}+\cdots +a_{m}b_{m}$; define M·a:=(M(1)·a,…,M(k)·a), where M(i) denotes the *i*-th row of M; and define $a\cdot M=(a\cdot M_{1},\ldots ,a\cdot M_{k})$, where $M_{j}$ denotes the *j*-th column of M.

We define the *span* $\langle U_{1},\ldots ,U_{n}\rangle$ of subspaces $U_{1},\ldots ,U_{n}$ of an ambient vector space *V* as the collection of all sums $u_{1}+\cdots +u_{n}$ with $u_{i}\in U_{i}$ for i∈[n]. (In other works, the span is sometimes denoted as $U_{1}+\cdots +U_{n}$.) We simply denote the span $\langle \langle u_{1}\rangle ,\ldots ,\langle u_{n}\rangle \rangle$ of the vectors $u_{1},\ldots ,u_{n}$ in *V* by $\langle u_{1},\ldots ,u_{n}\rangle$. We say that subspaces $U_{1},\ldots ,U_{n}$ of a vector space *V* are *independent* if $\dim\langle U_{1},\ldots ,U_{n}\rangle =\dim U_{1}+\cdots +\dim U_{n}$, where dimV denotes the *dimension* of a vector space *V*.

We repeatedly use *Grassmann’s identity*, which states that for vector spaces U,V we have

dimU∩V+dim〈U,V〉=dimU+dimV.

We need various notions from coding theory. For reference, see, e.g., [54].

The *support* supp(v) of a vector $v\in F_{q}^{n}$ is the collection of positions i∈{1,…,n} for which $v_{i}\neq 0$; the *(Hamming) weight* w(v) of v is the number of positions i∈{1,…,n} for which $v_{i}\neq 0$, that is, w(v)=|supp(v)|. The *(Hamming) distance* d(v,w) between $v,w\in F_{q}^{n}$ is the number of positions i∈{1,…,n} for which $v_{i}\neq w_{i}$. Note that d(v,w)=w(v−w).

A *code C* of length *n* over $F_{q}$ is just a subset of $F_{q}^{n}$; the code *C* is called *linear* if *C* is a *subspace* of $F_{q}^{n}$. We often refer to the vectors contained in a code as *codewords*. The *minimum weight* w(C) of a code *C* is the smallest weight of a nonzero codeword from *C*, and the *minimum distance* d(C) of *C* is the smallest distance between two distinct codewords from *C*. Note that if the code *C* is linear, then d(C)=w(C). We often refer to a linear code *C* of length *n*, dimension *k*, and minimum distance *d* over $F_{q}$ as an ${\left[n,k\right]}_{q}$ code or as an ${\left[n,k,d\right]}_{q}$ code; we simply write [n,k] or [n,k,d] if the intended field is clear from the context.

A *generator matrix* for an ${\left[n,k\right]}_{q}$ code *C* is a k×n matrix G over $F_{q}$ with rank *k* and with its rowspace equal to *C*, that is, *C* consists of the vectors a·G with $a\in F_{q}^{k}$. An (n−k)×n matrix H is a *parity-check matrix* for *C* if H has rank n−k and c∈C if and only if H·c=0. The *dual* code $C^{⊥}$ of *C* is the collection of all vectors x for which x·c=0 for all c∈C. It is not difficult to see that $C^{⊥}$ is an [n,n−k]-code, and has generator matrix H and parity-check matrix G, see also [54] (Chapter 11).

Finally, we need some notions related to MDS codes. As a general reference for this material, see [54] (Chapter 11). The *Singleton bound* states that an ${\left[n,k,d\right]}_{q}$ code satisfies d≤n−k+1. For a proof, see, e.g., [54] (Chapter 1, Theorem 11), or see [55] (Theorem 4.1) for a generalization for non-linear codes. An ${\left[n,k,n-k+1\right]}_{q}$ code, that is, a linear code that attains the Singleton bound, is called an *MDS code*. A related notion is that of an *arc*, a collection of nonzero vectors in $F_{q}^{k}$ with the property that any *k* of them are independent. (Usually, an arc is defined *projectively*, that is, as a set of points in PG(k−1,q), but for our purposes, this will do.) We say that a k×n matrix M represents an *n*-arc if the columns of M constitute an *n*-arc (i.e., an arc of size *n*) in $F_{q}^{k}$; alternatively, we refer to such a matrix as an *MDS-generator*. (The term *MDS matrix* comes from cryptography and is commonly reserved for a matrix *M* for which [IM] is an MDS-generator.) Consider an ${\left[n,k\right]}_{q}$ code *C*, with generator matrix G and parity-check matrix H. Obviously, if H has n−k columns that are dependent, then *C* has a nonzero codeword of weight at most n−k. Therefore, *C* is MDS if and only if the columns of H form an *n*-arc. Moreover, if G has *k* columns that are dependent, then there exists $a\in F_{q}^{k}$ with a≠0 such that the codeword $c=G^{⊤}a$ is nonzero but has a 0 in the corresponding positions, so that 0<w(c)≤n−k and *C* is not MDS. Hence, *C* is MDS if and only if G is an *n*-arc, that is, if and only if its generator matrix (or parity-check matrix) is an MDS-generator. In particular, *C* is MDS if and only if $C^{⊥}$ is MDS [56] and [57] (Lemma 6.7, p. 245).

Note that $F_{q}^{k}$ itself, the repetition codes with parameters ${\left[n,1,n\right]}_{q}$ and their duals, the codes with parameters ${\left[n,n-1,2\right]}_{q}$ (called even-weight codes when q=2), are all MDS codes. For k≥2, let m(k,q) denote the largest *n* for which an ${\left[n,k,n-k+1\right]}_{q}$ MDS code exists. The famous MDS conjecture, proven by Simeon Ball for the case where *q* is prime in [58], claims that

(2) $$m\left(k,q\right)=\left\{\begin{array}{cc} q+1, & \mathrm{for}\;2\leq k<q;\\ k+1, & \mathrm{for}\;k\geq q, \end{array}\right.$$

except that when *q* is even,

(3) m(3,q)=m(q−1,q)=q+2.

For k≥q, it was shown in [59] that m(k,q)=k+1, and that an ${\left[k+1,k\right]}_{q}$ MDS code is equivalent to the dual of the repetition code, see also [54] (Corollary 7). It is well known that m(k,q) is at least equal to the stated values in (2) and (3). Indeed, we already mentioned that

(4) $$\left(\begin{array}{ccccc} 1 & 0 & \cdots & 0 & -1\\ 0 & 1 & \cdots & 0 & -1\\ \vdots & \vdots & & \vdots & \vdots \\ 0 & 0 & \cdots & 1 & -1 \end{array}\right)$$

is an MDS-generator for all *k*; the corresponding linear code for q=2 is called the *even-weight code*. Furthermore, let $\alpha_{1},\ldots ,\alpha_{q-1}$ be the non-zero elements of $F_{q}$. If k≤q−1, then

(5) $$\left(\begin{array}{ccccc} 1 & \cdots & 1 & 1 & 0\\ \alpha_{1} & \cdots & \alpha_{q-1} & 0 & 0\\ \vdots & \cdots & \vdots & \vdots & \vdots \\ \alpha_{1}^{k-2} & \cdots & \alpha_{q-1}^{k-2} & 0 & 0\\ \alpha_{1}^{k-1} & \cdots & \alpha_{q-1}^{k-1} & 0 & 1 \end{array}\right)$$

is a k×(q+1) MDS-generator; moreover, if *q* is even, then

(6) $$\left(\begin{array}{cccccc} 1 & \cdots & 1 & 1 & 0 & 0\\ \alpha_{1} & \cdots & \alpha_{q-1} & 0 & 1 & 0\\ \alpha_{1}^{2} & \cdots & \alpha_{q-1}^{2} & 0 & 0 & 1 \end{array}\right)$$

is a 3×(q+2) MDS-generator. The corresponding codes are referred to as *(Generalized) Reed–Solomon codes*. In fact, for any *k*, 1≤k≤q+1, such that *q* is even or *k* is odd, there exists a [q+1,k,q−k+2] cyclic MDS code over $F_{q}$ [60] (this corrects an erroneous claim in [54]). For a reference for the above claims, see, e.g., [54] (Chapter 11, Sections 5–7).

## 3. Linear Storage Codes

In this paper, we adhere to the *vector space view* ([33,41,48,51,53,61,62,63]) on linear storage codes. Informally, a storage code with symbol alphabet $F_{q}$ is called *linear* if the four processes of data storage, data recovery, the generation of repair data from the helper nodes, and the generation of the replacement data from the repair data, are all linear operations over $F_{q}$ [29]. It turns out that in that case, the storage code can be described in terms of subspaces of an ambient vector space over $F_{q}$ referred to as the *message space*. In the description below, we will follow a similar approach as in [49,50]. We first need a few definitions.

**Definition** **1.** *We say that the subspaces $U_{1},\ldots ,U_{k}$ of a vector space V form a* recovery set *for V if $V=\langle U_{1},\ldots ,U_{k}\rangle$.*

**Definition** **2.** *We say that a subspace $U_{0}$ of a vector space V can be obtained from subspaces $U_{1},\ldots ,U_{r}$ of V by* β-repair*, written as*

$$U_{1},\ldots ,U_{r}\overset{\beta }{⟶}U_{0},$$

*if there are β-dimensional* helper *subspaces $H_{j}\subseteq U_{j}$ (j∈[r]) such that $U_{0}\subseteq \langle H_{j}|j\in \left[r\right]\rangle$.*

We can now present a formal definition of a Linear Regenerating Code (LRGC) in terms of vector spaces, which can be seen as a “basis-free” representation of a linear storage code. To understand the definition, think of the data that is stored by the storage code as being represented by a vector x in the ambient vector space $V≅F_{q}^{m}$, referred to as the *message space* of the code. Then for every subspace *W* of *V* that occurs in the definition, choose a fixed basis $w_{1},\ldots ,w_{t}$, and think of *W* as representing the *t* data symbols $x\cdot w_{1},\ldots ,x\cdot w_{t}$.

**Definition** **3.** *Let m,n,k,r,α,β be integers for which 1<k≤r<n and β≤α≤rβ. A linear storage code with parameters ${\left\{m,\left(n,k\right),\left(r,\alpha ,\beta \right)\right\}}_{q}$ consists of an ambient m-dimensional vector space V over $F_{q}$ together with a collection S of sequences $\sigma =U_{1},\ldots ,U_{n}$ of α-dimensional subspaces $U_{1},\ldots ,U_{n}$ of V, referred to as* coding states *of the storage code, with the following properties.*

*(i) (Data recovery) Every k subspaces in a coding state σ∈S constitute a recovery set for V. Moreover, we will assume that k is minimal with respect to this property.*

*(ii) (Repair) For every i∈[n] and for every J⊆[n]\{i} with |J|=r, there is a subspace $U_{i}^{\prime }$ of V such that ${\left(U_{j}\right)}_{j\in J}\beta U_{i}^{\prime }$ for which $\sigma^{\prime }:=U_{1},\ldots ,U_{i-1},U_{i}^{\prime },U_{i+1},\ldots ,U_{n}$ is again a coding state in S.*

For future use, we introduce some additional terminology.

**Definition** **4.** *We refer to the collection of all the α-dimensional subspaces of V that occur in some coding state in S as the* coding spaces *of the linear storage code S.*

*A subsequence $\pi =U_{1},\ldots ,U_{i-1},U_{i+1},\ldots ,U_{n}$ (i∈[n]) of a state $\sigma =U_{1},\ldots ,U_{n}\in S$ will be referred to as a* protostate *of the storage code S.*

So to actually employ the collection S as in Definition 3 as a storage code, think of the stored data as a vector x∈V (or as a *linear functional*, that is, as an element of the *dual* $V^{\vee }$ of *V* mapping a∈V to $x\cdot a\in F_{q}$ as in [64]). Then, for every coding space *U* involved in S, choose a *fixed* m×α matrix U=U(U) with columnspace equal to *U*; now, if *U* is the coding space associated with a particular storage node, then we let this node store the α symbols of the vector c(U,x):=x·U. Note that if u is any vector in *U*, with u=Ua∈U, say, then x·u=(x·U)·a, so for every u∈U, we can compute x·u from the stored vector x·U. Similarly, for a repair subspace *H* contained in a helper node with associated coding space *U* during repair, we choose a fixed m×β matrix H=H(H) with columnspace equal to *H*, and let this (helper) node contribute the β symbols x·H. The *code* associated with a coding state $\sigma =U_{1},\ldots ,U_{n}$ is the collection $C_{\sigma }$ of all words c(x) in $F_{q}^{n\alpha }$ obtained as the concatenation of the words $c(U_{i},x)$ for i∈[n] when x ranges over *V*. Note that $C_{\sigma }$ is an ${\left[n\alpha ,m\right]}_{q}$ code with m×nα generator matrix

$$G\left(\sigma \right)=\left[U_{1}\cdots U_{n}\right],$$

where $U_{i}$ is a matrix with columnspace $U_{i}$, for all *i*. It is not difficult to verify that the family of codes $C_{\sigma }$ associated with states σ from a storage code S as in Definition 3 indeed has the desired repair properties when used in this way to store data. Note that the resulting functional-repair (FR) storage code is exact-repair precisely when the code consists of a *single* coding state. In the case where the storage code is FR, at any time every storage node must “know” its associated coding space. The extra overhead that this entails can be relatively small if the code is used to store a *large number* of data vectors *simultaneously*. For further details, we refer to [49,50]. The next example illustrates the above.

**Example** **1** (See also [48] (Example 2.2), [49] (Example 2.6), and [50] (Example 2.7))**.** *We will construct a binary linear functional-repair storage code S with parameters ${\left\{m,\left(n,k\right),\left(r,\alpha ,\beta \right)\right\}}_{q}={\left\{5,\left(4,3\right),\left(3,2,1\right)\right\}}_{2}$ (representing the smallest non-MSR/MBR extreme point of the achievable cut-set region). So let V be a 5-dimensional vector space over $F_{2}$. A set of three 2-dimensional subspaces $\left\{U_{1},U_{2},U_{3}\right\}$ of V is said to be* (3,2)-regular *if any two of them are independent and $\langle U_{1},U_{2},U_{3}\rangle =V$ (this was called* (3,1)-good *in the cited papers). It is easily verified that if $\left\{U_{1},U_{2},U_{3}\right\}$ is (3,2)-regular, then there are nonzero vectors $a_{i}\in U_{i}$ (i=1,2,3) such that $a_{1}+a_{2}+a_{3}=0$; as a consequence, there is a basis $e_{1},e_{2},e_{3},a_{1},a_{2}$ for V such that $U_{i}=\langle e_{i},a_{i}\rangle$ (i=1,2,3). It is easily checked that with $U_{4}:=\langle e_{1}+e_{2},e_{1}+e_{3}\rangle$, any subset of $\left\{U_{1},U_{2},U_{3},U_{4}\right\}$ of size 3 is (3,2)-regular. As a consequence, the collection of all states $\sigma =U_{1},U_{2},U_{3},U_{4}$ for which any set of three of the spaces form a (3,2)-regular collection is a linear storage code with the parameters as specified. Note that there are coding states that are* unreachable*, that is, not obtainable by repair from a protostate; for example, states of the form $\sigma =U_{1},U_{2},U_{3},U_{4}$ with $U_{i}=\langle e_{i},a_{i}\rangle$ (i=1,2,3) and with $U_{4}=\langle e_{1}+e_{2},e_{1}+e_{3}+a_{1}\rangle$; obviously, such states can be freely deleted from the code.*

## 4. The Cut-Set Bound Revisited

Suppose that the DSS employs an {m,(n,k),(r,α,β)} storage code. Since *k* is assumed to be minimal and any *r* nodes can regenerate the stored information, we have n−1≥r≥k. (Indeed, to see this, choose an arbitrary set of *r* helper nodes, and one by one destroy and repair all the other nodes, employing these helper nodes for each repair. Then the information contained in the system is just the information that is contained in these *r* helper nodes.) Note also that, obviously, m/k≤α (since any *k* nodes regenerate the stored information), and α≤rβ (since *r* helper nodes, each contributing an amount β of information, can create a replacement node), and β≤α (since α is the maximum amount that can be contributed by a helper node). Finally, let *s* be an integer such that (s−1)β≤α≤sβ, or such that β≤α≤sβ if s=r−k+1; therefore, we may assume that r−k+1≤s≤r. We let $\bar{\alpha }:=\alpha /m$ and $\bar{\beta }:=\beta /m$ denote the *normalized* storage capacity and transport capacity, respectively. Our aim is to provide a quick and informal derivation of the cut-set bound for RGCs and to establish a few simple properties of optimal codes that seem to have gone unobserved. First, we show the following.

**Lemma** **1** (Cut-set bound**).** *Let m,n,k,r be positive integers with n−1≥r≥k, and let α,β be positive real numbers with β≤α≤rβ. Let s be an integer such that (s−1)β≤α≤sβ if s=r−k+2,…,r or such that β≤α≤(r−k+1)β if s=r−k+1. A storage code with parameters {m,(n,k),(r,α,β)} satisfies*

(7) $$m\leq \sum_{i=0}^{k-1}\min\left(\alpha ,\left(r-i\right)\beta \right)=\left(r-s+1\right)\alpha +\left(\left(s-1\right)+\cdots +\left(r-k+1\right)\right)\beta .$$

*Moreover, in the case of equality in (7), we have the following.*

- *Any r−s+1 nodes, together, contain an amount of information (r−s+1)α, that is, these nodes carry independent information.*
- *Any two nodes carry an amount of information of at least 2α if s<r or α+(k−1)β if s=r. Therefore, if k=1, then every node carries the stored information, so the code is essentially a repetition code, but if k≥2, then no two nodes carry identical information .*
- *If, in addition, we have r=k, then for any J⊆[n] with size |J|≤k, the information $I(N_{j}|j\in J)$ contained in any collection of storage nodes $N_{j}$ with j∈J satisfies*
(8) $$I(N_{j}|j\in J)=|J|\alpha$$
  *if |J|≤r−s+1, and*
(9) $$I\left(N_{j}|j\in J\right)\geq \left(r-s+1\right)\alpha +\left(\left(s-1\right)+\cdots +\left(s-t\right)\right)\beta .$$
  *if |J|=r−s+1+t with 1≤t≤k−r+s−1.*

**Proof.**  Assume that nodes $N_{1},\ldots ,N_{n}$ store the file, and that each *k* nodes regenerate the stored file, with every node storing α symbols. Consider nodes $N_{1},\ldots ,N_{r+1}$. Pretend that nodes $N_{r-s+2},\cdots ,N_{k}$ fail in turn, and are replaced by newcomer nodes $N_{r-s+2}^{\prime },\ldots ,N_{k}^{\prime }$, with none of the nodes $N_{r+2},\ldots ,N_{n}$ ever participating in a repair. Assume that for i=1,…,k−r+s−1, the lost node $N_{r-s+1+i}$ is replaced by newcomer node $N_{r-s+1+i}^{\prime }$, which receives an amount of β information from each node contained in the set of *r* helper nodes consisting of the old nodes $N_{1},\ldots ,N_{r-s+1}$, the new nodes $N_{r-s+2}^{\prime },\ldots ,N_{r-s+i}^{\prime }$, and the old nodes $N_{r-s+2+i},\ldots ,N_{r+1}$. Now consider the sequence K of *k* nodes defined by $K:=N_{1},\ldots ,N_{r-s+1},N_{r-s+2}^{\prime },\ldots ,N_{k}^{\prime }$. The first r−s+1 nodes $N_{1},\ldots ,N_{r-s+1}$ in K contain an amount of information that is at most equal to (r−s+1)α. And for i=1,…,k−r+s−1, the information in $N_{r-s+1+i}^{\prime }$ that is not already contained in the preceding nodes $N_{1},\ldots ,N_{r-s+1},N_{r-s+2}^{\prime },\ldots ,N_{r-s+i}^{\prime }$ in K is the information obtained from $N_{r-s+2+i},\ldots ,N_{r+1}$, so is at most equal to (s−i)β. As a consequence, the amount of information contained in K is at most equal to (r−s+1)α+(1+2+⋯+(r−k+1))β, and since any *k* nodes should be able to regenerate the stored information, we conclude that (7) holds. Moreover, we conclude that if the bound (7) holds with equality, then the nodes $N_{1},\ldots ,N_{r-s+1}$ in K, together, contain an amount of (r−s+1)α of information, and, in addition, a node $N_{r-s+2+i}$ contributes a further amount iβ of information that is independent of the information already present in preceding nodes in K.

By keeping track which of the nodes among $N_{r-s+2},\ldots ,N_{r+1}$ contributed the various pieces of information during the above repair process, we see that node $N_{r-s+2+i}$ for i=1,…,k−r+s−2 contributes an independent amount of information iβ, and the nodes $N_{k+1},\ldots ,N_{r+1}$ each contribute an independent amount (k−r+s−1)β. Also note that the sequence of nodes $N_{1},\ldots ,N_{k}$, as well as their order, is arbitrary, and nodes $N_{1}$ and $N_{r+1}$ form an arbitrary pair of nodes. Now, if s<r, then r−s+1≥2 and we already showed that any r−s+1 nodes, together, contain at least an amount of 2α of information; and if s=r then nodes $N_{1}$ and $N_{r+1}$, together, contain at least an amount of α+(k−r+s−1)β=α+(k−1)β of information. Obviously, in the case where k=1, every node carries the same information, so the code is essentially a repetition code. Finally, in the case where r=k, by considering the sequence of nodes $N_{1},\ldots ,N_{r-s+1},N_{r+1},\ldots ,N_{r-s+3}$, we see that the last claim in the lemma holds.  □

**Definition** **5.** *We say that a Regenerating Code (RGC) with parameters {m,(n,k),(r,α,β)} is* optimal *if the bound (1) is attained with equality, and if, moreover, lowering α or β results in violation of this bound.*

Note that if α≤(r−k+1)β, then (7) reads as m≤kα. In that case, if the code is optimal, then according to Definition 5, we must have α=m/k and β=α/(r−k+1).

It is not difficult to see that in terms of the normalized parameters $\bar{\alpha }:=\alpha /m$ and $\bar{\beta }:=\beta /m$, we have the following. For s∈{r−k+1,…,r}, define

(10) mk,r,s:=(r−s+1)s+(s−1)+⋯+(r−k+1)=(r−s+1)s+s2−r−k+12,

and set

(11) $${\bar{\alpha }}_{s}:=s/m_{k,r,s},\;{\bar{\beta }}_{s}:=1/m_{k,r,s}.$$

Then the *feasible cut-set region*, the region of all pairs $\left(\bar{\alpha },\bar{\beta }\right)$ that can be realized by tuples (m,k,r,α,β) for which m≤kα, β≤α≤rβ, and for which (7) holds with *s* as defined above, has extreme points $\left({\bar{\alpha }}_{s},{\bar{\beta }}_{s}\right)$ for s=r−k+1,…,r, and is further bounded by the half-lines $\bar{\alpha }=1/k={\bar{\alpha }}_{r-k+1},\bar{\beta }\geq \bar{\alpha }/\left(r-k+1\right)={\bar{\beta }}_{r-k+1}$ and $r\bar{\beta }=\bar{\alpha }\geq 2/\left(2rk-k^{2}+k\right)$, see Figure 1 in Section 1.

We sometimes refer to the extreme points $\left({\bar{\alpha }}_{s},{\bar{\beta }}_{s}\right)$ (s=r−k+1,…,r) as the *corner points* of the achievable region. The corner points $\left({\bar{\alpha }}_{r-k+1},{\bar{\beta }}_{r-k+1}\right)$ and $\left({\bar{\alpha }}_{r},{\bar{\beta }}_{r}\right)$ are known as the *MSR point* and the *MBR point*, respectively (note that these points are equal if and only if k=1).

**Definition** **6.** *We say that an RGC with parameters $\{m,\left(n,k\right),{\left(r,\alpha ,\beta \right\}}_{q}$* attains a corner point *of the achievable cut-set region if the pair (α/m,β/m) equals one of the pairs $\left({\bar{\alpha }}_{s},{\bar{\beta }}_{s}\right)$ with s∈{r−k+1,…,r}. An RGC that attains the MSR point or the MBR point is referred to as an* MSR code *or an* MBR code*, respectively.*

**Remark** **1.** *The result in (9) may well hold also for optimal storage codes where r>k, but we have no proof and no counterexample.*

**Remark** **2.** *There are cases of optimal codes where (9) is not satisfied with equality. Consider an MBR code with α=r=k=3, n=4, and m=3+2+1=6. The “standard” code has coding spaces $U_{i}=\langle e_{\left\{i,j\right\}}|j\in \left[4\right],j\neq i\}$, where the C(4,2)=6 vectors $e_{\left\{i,j\right\}}$ with 1≤i<j≤4 form a basis. This code satisfies (8) and (9) with equality.*

*Now, let $U_{1}=\langle e_{1},e_{2},e_{3}\rangle$, $U_{2}=\langle e_{4},e_{5},e_{6}\rangle$, $U_{3}=\langle e_{1}+e_{4},e_{2},e_{6}\rangle$, and $U_{4}=\langle e_{2}+e_{6},e_{3},e_{5}\rangle$. Note that $U_{4}$ can be obtained by repair from $U_{1}$ (use $e_{3}$), $U_{2}$ (use $e_{5}$), and $U_{3}$ (use $e_{2}+e_{6}$). Now any two coding spaces span at least a 5-space, and any three span a 6-space, but $U_{1},U_{2}$ are independent.*

*This example shows that in a coding state, (9) is not necessarily satisfied with equality. But note that this example can only represent an* unreachable *state in a storage code with these parameters, since once we have a protostate with no two spaces disjoint, then the new space has a repair vector in common with each of the other coding spaces.*

## 5. (k,r,s,β)-Regular Configurations

In this section, let n,k,r be integers with n−1≥r≥k≥1, let *s* be an integer with r−k+1≤s≤r, and let $m_{k,r,s}$ be as defined in (10). Moreover, let β be a positive integer and let α:=sβ. Motivated by the results from the previous section—notably, by (8) and (9))—and by the form of the “small” storage codes from [48,49]) (see also [50]), we introduce and investigate the following notion.

**Definition** **7.** *Let V be a vector space with $\dim V=m_{k,r,s}\beta$, and let $U_{1},\ldots ,U_{n}$ be α-dimensional subspaces of V. We say that the collection $\left\{U_{1},\ldots ,U_{n}\right\}$ is* (k,r,s,β)-regular *in V if α=sβ and, for every integer t with 0≤t≤k−(r−s+1) and for every J⊆[n] with |J|=r−s+1+t, we have $\dim\langle U_{j}|j\in J\rangle =d_{t}\beta$, where* 

(12) $$d_{t}:=d_{t}^{r,s}:=\left(r-s+1\right)s+\sum_{i=1}^{t}\left(s-i\right)=\left(r-s+1\right)s+\left(s-1\right)+\cdots +\left(s-t\right).$$

*In addition, we say that $\left\{U_{1},\ldots ,U_{n}\right\}$ is* (k,r,s)-regular *if it is (k,r,s,β)-regular with β=1, and (r,s)-regular if it is (k,r,s)-regular with k=r. We will write* 

(13) $$m_{r,s}:=m_{r,r,s}=\left(r-s+1\right)s+\left(s-1\right)+\cdots +1$$

*to denote the dimension of the ambient space of an (r,s)-regular collection.*

Note that Definition 7 requires, in particular, that any r−s+1 of the vector spaces in a (k,r,s,β)-regular collection are independent, and that any *k* of the vector spaces span *V*. Our aim in the remainder of this section is to study the properties of the numbers $m_{k,r,s}$ defined in (10), and to describe a construction of (k,r,s)-regular collections (and, hence, of (k,r,s,β)-regular configurations for all integers β). To that end, we need the following.

**Lemma** **2.** *For i∈[s], define $m_{i}:=\min\left(r-s+i,k\right)$. Then $r-s+1=m_{1}\leq \cdots \leq m_{s}=k$. Let t be an integer with 0≤t≤k−(r−s+1), and set u:=(r−s+1)+t. Then r−s+1≤u≤k and*

$$d_{t}=\left(r-s+1\right)s+\left(s-1\right)+\cdots +\left(s-t\right)=\sum_{i=1}^{s}\min\left(m_{i},u\right).$$

*In particular, for $m_{k,r,s}$ as defined in (10), we have*

$$m_{k,r,s}=d_{s-(r-k+1)}=m_{1}+m_{2}+\cdots +m_{s}.$$

**Proof.**  Since r−k+1≤s, we have r−s+1≤k, hence $m_{1}=r-s+1$. Also, $m_{s}=\min\left(r,k\right)=k$. Obviously, $m_{i}\leq m_{j}$ if i<j. Therefore, the first claim follows immediately. Since u=(r−s+1)+t≤k, we have $\min\left(m_{i},u\right)=\min\left(r-s+i,u\right)$, so we have

$$\begin{array}{ccc} \sum_{i=1}^{s}\min\left(m_{i},u\right) & = & (r-s+1)+\cdots +(r-s+t)+(s-t)u\\ & = & (r-s+1)t+0+1+\cdots +(t-1)+(s-t)(r-s+1)+(s-t)t\\ & = & \left(r-s+1)s+0+1+2+\cdots +(t-1)+t(s-t\right)\\ & = & \left(r-s+1\right)s+\left(s-t\right)+\cdots +\left(s-1\right)=d_{t}. \end{array}$$

Taking t=k−(r−s+1), we have $u=k\geq m_{i}$ for all *i*, and we find that $m_{k,r,s}=d_{k-(r-s+1)}=\sum_{i=1}^{s}\min\left(m_{i},k\right)=\sum_{i=1}^{s}m_{i}$.  □

Now, to construct a (k,r,s)-regular configuration of size n≥r+1, we proceed as follows. For i∈[s], let $M_{i}$ be a $m_{i}\times r$ MDS-generator over a sufficiently large field $F_{q}$, and let $M:=\mathrm{diag}(M_{1},\ldots ,M_{s})$. Now let $U_{j}:=\langle M_{1}\left(j\right),M_{2}\left(j\right),\ldots ,M_{s}\left(j\right)\rangle$, where $M_{i}\left(j\right)$ denotes the *j*-th column of $M_{i}$. Also, write $V_{i}^{\prime }=F_{q}^{m_{i}}$ and let $V:=V_{1}⊕\cdots ⊕V_{s}=\langle V_{1},\ldots ,V_{s}\rangle$, where we identify $V_{i}^{\prime }$ with the subspace $V_{i}:=\left\{0\right\}⊕\cdots ⊕\left\{0\right\}⊕V_{i}^{\prime }⊕\left\{0\right\}⊕\cdots ⊕\left\{0\right\}$ of *V*. Note that $\dim U_{j}=s$ (j∈[n]), and, by Lemma 2, we have that $\dim V=m_{k,r,s}$.

**Theorem** **1.** *Given the above definitions, $\sigma :=\{U_{1},\ldots ,U_{n}\}$ is (k,r,s)-regular, and σ can be constructed from a generator matrix of an ${\left[r,k,r-k+1\right]}_{q}$ MDS code (that is, from a k×r MDS-generator).*

**Proof.**  We begin by remarking that since $m_{i}\leq k\leq r$ and $m_{s}=k$, the matrices $M_{i}$ can indeed be constructed if the field size *q* is large enough. Indeed, the matrices $M_{1},\ldots ,M_{s-1}$ can be constructed from a matrix $M_{s}$ by deleting some columns, and since $m_{s}=k$, such a matrix exists if and only if there exists an ${\left[r,k,r-k+1\right]}_{q}$ MDS code. Note that for i∈[s], the columns of $M_{i}$ are in $V_{i}^{\prime }$; hence, the corresponding columns in *M* are in $V_{i}$. Next, consider the span of a collection $U_{i}$ for i∈I, where |I|=u. Since this span contains *u* vectors from $V_{i}$, which correspond to *u* columns from $M_{i}$, the MDS property of $M_{i}$ implies that the dimension of their span is equal to $\min(m_{i},u)$. Therefore, with u:=(r−s+1)+t, according to Lemma 2, the span in *V* is equal to $\sum_{i=1}^{s}\min\left(m_{i},u\right)=d_{t}$, as required. In particular, for t:=k−(r−s+1), we have $m:=\dim V=d_{t}=m_{k,r,s}$.  □

The above suggests investigating storage codes with parameters $\left\{m_{k,r,s},\left(n,k\right),\left(r,s,1\right)\right\}$ and with coding states that are (k,r,s)-regular. This is the subject of Section 7 and Section 8 for the case where k=r. We note that not every such coding state is reachable by repair, see Example 2 below.

**Example** **2.** *Let $U_{1}:=\langle a_{1},b_{1}\rangle$. $U_{2}:=\langle a_{2},b_{2}\rangle$, $U_{3}:=\langle a_{3},b_{1}+b_{2}\rangle$, and $U_{4}:=\langle -a_{1}-a_{2}-a_{3},b_{1}-b_{2}\rangle$, where $V:=\langle a_{1},a_{2},a_{3},b_{1},b_{2}\rangle$ has dimension 5. Then $\sigma :=\{U_{1},U_{2},U_{3},U_{4}\}$ is (3,2)-regular, but no subspace $U_{i}$ can be obtained from the other three subspaces $U_{j}$ with j≠i by 1-repair. Therefore, σ cannot be a reachable coding state in a {5,(4,3),(3,2,1)} storage code. Replacing $U_{4}$ by $U_{4}^{\prime }:=\langle a_{1}-a_{2},a_{1}-a_{3}\rangle$ yields a (3,2)-regular configuration that could be a reachable state in a storage code with these parameters.*

In Section 8, we shall describe an alternative construction of an (r,s)-regular configuration. Here, we state a useful property of the numbers $m_{k,r,s}$ that is needed in that construction.

**Lemma** **3.** *We have*

$$\begin{array}{ccc} m_{k,r,s} & = & \left(r-s+1)s+(s-1)+\cdots +(r-k+1\right)\\ & = & \left\{\begin{array}{cc} r+m_{k-1,r-1,s-1}, & if\;s>r-k+1);\\ ks, & if\;s=r-k+1, \end{array}\right. \end{array}$$

*and hence*

(14) $$m_{k,r,s}=r+\left(r-1\right)+\cdots +\left(2r-s-k+2\right)+\left(r-s+1\right)\left(r-k+1\right).$$

**Proof.**  If r−k+1<s, then with $r^{\prime }:=r-1,k^{\prime }:=k-1,s^{\prime }:=s-1$, we have

$$\begin{array}{ccc} m_{k,r,s} & = & \left(r-s+1)s+(s-1)+\cdots +(r-k+1\right)\\ & = & \left(r^{\prime }-\alpha^{\prime }+1\right)s^{\prime }+\left(r-s^{\prime }\right)+s^{\prime }+\left(s^{\prime }-1\right)+\left(r^{\prime }-k^{\prime }+1\right)\\ & = & r+m_{k^{\prime },r^{\prime },s^{\prime }}. \end{array}$$

The last claim follows immediately from this claim by induction.  □

## 6. The Structure of an (r,r,s,β)-Regular Configuration

In this section, we consider the case where r=k. We begin with a result that is fundamental for what follows.

**Lemma** **4.** *Let $U_{1},\ldots ,U_{r}$ be subspaces of a vector space V. Define*

(15) $${\bar{U}}_{i}:=\langle U_{j}|j\in \left[r\right],j\neq i\rangle .$$

*Suppose that $H_{i}$ is a subspace of $U_{i}$ with $H_{i}\cap {\bar{U}}_{i}=\left\{0\right\}$ for all i∈[r]. Then, with $H:=\langle H_{i}|i\in \left[r\right]\rangle$, we have $\dim H=\sum \dim H_{i}$, and for every J⊆[r], we have $\langle U_{j}|j\in J\rangle \cap H=\langle H_{j}|j\in J\rangle$.*

**Proof.**  Let *j* and *t* be integers with 0≤j<t≤r. Since $U_{1},\ldots ,U_{j},H_{1},\ldots ,H_{t-1}\subseteq {\bar{U}}_{t}$ and $H_{t}\cap {\bar{U}}_{t}=\left\{0\right\}$, we have $\dim\langle U_{1},\ldots ,U_{j},H_{1},\ldots ,H_{t}\rangle =\dim\langle U_{1},\ldots ,U_{j},H_{1},\ldots ,H_{t-1}\rangle +\dim H_{t}$. Since $H_{1},\ldots ,H_{j}\subseteq \langle U_{1},\ldots ,U_{j},\rangle$, by induction we have that

(16) $$\dim\langle U_{1},\ldots ,U_{j},H_{1},\ldots ,H_{r}\rangle =\dim\langle U_{1},\ldots ,U_{j}\rangle +\sum_{i=j+1}^{r}\dim H_{i}.$$

By (16) for j=0, we conclude that $\dim H=\sum \dim H_{i}$, which proves the first part of the lemma. Next, let J⊆[r] with |J|=j. After renumbering the subspaces if necessary, we may assume that J={1,…,j}. By (16) and Grassmann’s identity, we have

$$\dim\langle U_{1},\ldots ,U_{j}\rangle \cap H=\dim\langle U_{1},\ldots ,U_{j}\rangle +\dim H-\dim\langle U_{1},\ldots ,U_{j},H\rangle =\sum_{i=1}^{j}\dim H_{j}.$$

Since $H_{1},\ldots ,H_{j}\subseteq \langle U_{1},\ldots ,U_{j}\rangle$ and $\dim\langle H_{1},\ldots ,H_{j}\rangle =\sum_{i=1}^{j}\dim H_{i}$, we conclude that $\langle U_{1},\ldots ,U_{j}\rangle \cap H=\langle H_{1},\ldots ,H_{j}\rangle$, so the second part of the lemma follows.  □

Now assume that *r*, *s*, and β are positive integers with 1≤s≤r and r≥2; set α:=sβ; and let *V* be an *m*-dimensional vector space over some finite field $F_{q}$ with $m=m_{r,s}\beta$, where $m_{r,s}$ is as defined in (10). Assume that $\pi =\{U_{1},\ldots ,U_{r}\}$ is (r,r,s,β)-regular in *V*. For i∈[r], let $H_{i}$ be a β-dimensional subspace of $U_{i}$ with $H_{i}\cap {\bar{U}}_{i}=\left\{0\right\}$, where ${\bar{U}}_{i}$ is as defined in (15), and define $H:=\langle H_{1},\ldots ,H_{r}\rangle$. Below, we will use these assumptions to draw a number of conclusions. First note that since π is (r,r,s,β)-regular, we have

(17) $$\dim{\bar{U}}_{i}=m-\beta$$

and

(18) $$\langle {\bar{U}}_{i},U_{i}\rangle =V$$

for all i∈[r]. By Lemma 4, $H_{1},\ldots ,H_{r}$ are independent in *H*, so dimH=rβ. Next, we note the following.

**Lemma** **5.** *We have that*

$$\dim{\bar{U}}_{1}\cap {\bar{U}}_{2}\cap \cdots \cap {\bar{U}}_{t}=m-t\beta$$

*for all t; in particular, with*

(19) $$V^{\prime }:=\cap_{j=1}^{r}{\bar{U}}_{j},$$

*we have $\dim V^{\prime }=m-r\beta$.*

**Proof.**  We use induction on *t*. By (17), the result certainly holds for t=1. Now, let t≥2, and suppose the claim holds for smaller values of *t*. First, we observe that since $U_{t}$ is contained in ${\bar{U}}_{1},\ldots ,{\bar{U}}_{t-1}$, by (18), we have $\langle {\bar{U}}_{1}\cap \cdots \cap {\bar{U}}_{t-1},{\bar{U}}_{t}\rangle ⊇\langle U_{t},{\bar{U}}_{t}\rangle =V$. Hence

(20) $$\dim\langle {\bar{U}}_{1}\cap \cdots \cap {\bar{U}}_{t-1},{\bar{U}}_{t}\rangle =m.$$

By the induction hypothesis, $\dim{\bar{U}}_{1}\cap \cdots \cap {\bar{U}}_{t-1}=m+\left(t-1\right)\beta$, so using (17), (20), and Grassmann’s identity, we obtain

$$\begin{array}{ccc} \dim{\bar{U}}_{1}\cap \cdots \cap {\bar{U}}_{t} & = & \dim{\bar{U}}_{1}\cap \cdots \cap {\bar{U}}_{t-1}+\dim{\bar{U}}_{t}-\dim\langle {\bar{U}}_{1}\cap \cdots \cap {\bar{U}}_{t-1},{\bar{U}}_{t}\rangle \\ & = & (m-(t-1)\beta )+(m-\beta )-m=m-t\beta . \end{array}$$

The last claim in the lemma follows by letting t=r.  □

**Lemma** **6.** *We have $\langle V^{\prime },H\rangle =V$ and $V^{\prime }\cap H=\left\{0\right\}$. (We will write this as $V=V^{\prime }⊕H$, identifying $V^{\prime }$ with $V^{\prime }⊕\left\{0\right\}$ and H with {0}⊕H.)*

**Proof.**  We already noted that dimH=rβ. Moreover, since r≥2, using Lemma 4 we have

$$H\cap V^{\prime }\subseteq H\cap {\bar{U}}_{1}\cap {\bar{U}}_{2}=\left(H\cap {\bar{U}}_{1}\right)\cap \left(H\cap {\bar{U}}_{2}\right)=H_{1}\cap H_{2}=\left\{0\right\}.$$

By Lemma 5, we have $\dim V^{\prime }=m-r\beta$, so $\dim V=\dim V^{\prime }+\dim H$, and the claimed result follows.  □

Next, for i=1,…,r, we define

(21) $$U_{i}^{\prime }:=U_{i}\cap V^{\prime }.$$

**Lemma** **7.** *For all i∈[r], we have $\dim U_{i}^{\prime }=\left(s-1\right)\beta$ and $U_{i}=U_{i}^{\prime }⊕H_{i}$.*

**Proof.**  Let i∈[r]. Since $U_{i}\subseteq {\bar{U}}_{j}$ for j≠i, we have that

$$U_{i}^{\prime }=U_{i}\cap V^{\prime }=U_{i}\cap \left(\cap_{j=1}^{r}{\bar{U}}_{j}\right)=U_{i}\cap {\bar{U}}_{i}.$$

So by (17), (18), and Grassmann’s identity, we have

$$\dim U_{i}^{\prime }=\dim U_{i}\cap {\bar{U}}_{i}=\dim U_{i}+\dim{\bar{U}}_{i}-\dim\langle U_{i},{\bar{U}}_{i}\rangle =s\beta +\left(m-\beta \right)-m=\left(s-1\right)\beta .$$

Since $U_{i}^{\prime },H\subseteq U_{i}$, $U_{i}^{\prime }=U_{i}\cap V^{\prime }$, and $H\cap U_{i}=H_{i}$ by Lemma 4, the claimed results now follow.  □

We summarize the above result in the following theorem.

**Theorem** **2.** *Let r, s, and β be positive integers with 2≤s≤r and r≥2; set α:=sβ; and let V be a vector space with $m:=\dim V=m_{r,s}\beta$, with $m_{r,s}$ as defined in (13).*

*(i) Let $V^{\prime }$ and H be subspaces of V for which $V=\langle V^{\prime },H\rangle$ and $V^{\prime }\cap H=\left\{0\right\}$ (so that $V=V^{\prime }⊕H$), and let $m^{\prime }:=\dim V^{\prime }=m-r\beta =m_{r-1,s-1}$ and dimH=rβ. Furthermore, let $H_{1},\ldots ,H_{r}$ be independent in H with $\dim H_{i}=\beta$ (i∈[r]), and let $\sigma^{\prime }=\left\{U_{1}^{\prime },\ldots ,U_{r}^{\prime }\right\}$ be (r−1,r−1,s−1,β)-regular in $V^{\prime }$. Then, with $U_{i}:=\langle U_{i}^{\prime },H_{i}\rangle =U_{i}^{\prime }⊕H_{i}$ (i∈[r]), we have that $\pi =\{U_{1},\ldots ,U_{r}\}$ is (r,r,s,β)-regular in V; moreover, $V^{\prime }$ satisfies (19), $U_{i}^{\prime }=U_{i}\cap V^{\prime }$, $H_{i}\subseteq U_{i}$, and $H_{i}\cap {\bar{U}}_{i}=\left\{0\right\}$, where ${\bar{U}}_{i}$ is as defined in (15).*

*(ii) Conversely, if $\pi =\{U_{1},\ldots ,U_{r}\}$ is (r,r,s,β)-regular in V, then π can be put in the form as in (i) by letting $V^{\prime }$ be as in (19), and, for all i∈[r], letting $U_{i}^{\prime }:=U_{i}\cap V$ and choosing $H_{i}\subseteq U_{i}$ with $H_{i}\cap {\bar{U}}_{i}=\left\{0\right\}$.*

**Proof.**  We first note that $m-r\beta =m_{r-1,s-1}$ by Lemma 3. With $d_{t}^{r,s}$ as in (12), we have $d_{t}^{r,s}=d_{t}^{r-1,s-1}+\left(r-s+1\right)+t$ for integers *t* with 0≤t≤s−2. Now, if $U_{i}=U_{i}^{\prime }⊕H_{i}$ (i∈[r]), then with J⊆[r] with |J|=r−s+1+t and 0≤t≤s−1, we have $\dim\langle U_{j}|j\in J\rangle =\dim\langle U_{j}^{\prime }|j\in J\rangle +\beta \left|J\right|$. So for t<s−1, we have $\dim\langle U_{j}|j\in J\rangle =d_{t}^{r,s}\beta$ if and only if $\dim\langle U_{j}^{\prime }|j\in J\rangle =d_{t}^{r-1,s-1}\beta$, and, in addition, $\dim\langle U_{j}|j\in \left[r\right]\rangle =\dim V$ if and only if $\dim\langle U_{j}^{\prime }|j\in \left[r\right]\rangle =\dim V^{\prime }$. We conclude that π is (r,r,s,β)-regular in *V* if and only if $\sigma^{\prime }$ is (r−1,r−1,s−1,β)-regular in $V^{\prime }$. This proves part (i); part (ii) follows from Lemmas 5–7.  □

The next lemma handles the case where s=1.

**Lemma** **8.** *Let $\sigma =\{U_{1},\ldots ,U_{r+1}\}$ be (r,r,1,β)-regular in a vector space V with $m:=\dim V=m_{r,1,\beta }\beta =r\beta$. Then there is a basis $\left\{h_{i,j}|i\in \left[r\right],j\in \left[\beta \right]\right\}$ of V such that $U_{i}=\langle h_{i,j}|j\in \beta \rangle$ for i∈[r] and $U_{r+1}=\langle -h_{1,j}-\cdots -h_{r,j}|j\in \left[\beta \right]\rangle$. In particular, the resulting storage code is linear, exact-repair, and optimal, meeting the cut-set bound in the MSR point.*

**Proof.**  Since σ is (r,r,1,β)-regular, $U_{1},\ldots ,U_{r}$ are independent in *V* and every vector u in $U_{r+1}$ is of the form $u=u_{1}+\cdots +u_{r}$ with $u_{i}\in U_{i}$ (i∈[r]). Now, let $h_{1},\ldots ,h_{\beta }$ be a basis for $U_{r+1}$, and let $h_{i}=h_{i,1}+\cdots +h_{i,\beta }$ with $h_{i,j}\in U_{i}$ for j∈[β] and i∈[r]. Since $\langle U_{j}|j\in \left[r+1\right],j\neq i\rangle =V$, we conclude that $U_{i}=\langle h_{i,j}|j\in \left[\beta \right]\rangle$ for all i∈[r]. Since $U_{r+1}=\langle -h_{1},\ldots ,-h_{r}\rangle$, the first claim follows. It is also easily checked that a lost coding space $U_{i}$ can be exactly repaired from knowledge of all the vectors $h_{t,j}$ (j∈[β] for t∈[r+1], t≠i. Since s=1, the resulting code is an ER MSR storage code.  □

The case where s=r is more complicated, as is illustrated by the example below.

**Example** **3.** *The standard example is the following. Let dimV=β(r+1)r/2, let $H_{\left\{i,j\right\}}$ (1≤i<j≤r+1 be independent in V with $\dim H_{\left\{i,j\right\}}=\beta$, and let $U_{i}=\langle H_{\left\{i,j\right\}}|j\in \left[r+1\right],j\neq i\rangle$. Then $\sigma =\{U_{1},\ldots ,U_{r+1}\}$ is (r,r,r,β)-regular in V. But already for r=2 and β=1 we have a different example. Indeed, let $\dim V=m_{2,2}=3$ with $V=\langle e,a_{1},a_{2}\rangle$, and let $U_{1}:=\langle e,a_{1}\rangle$, $U_{2}:=\langle e,a_{2}\rangle$, and $U_{3}:=\langle e,a_{1}+a_{2}\rangle$. Then $\sigma =\{U_{1},U_{2},U_{3}\}$ is (2,2)-regular.*

We leave the determination of (r,r,r,β)-regular configurations as an open problem.

## 7. Main Results

In this section, we specialize to the case where β=1 and, except in Corollary 1, also k=r. The following simple result may be of independent interest.

**Lemma** **9.** *Let $U_{1},\ldots ,U_{r}$ be subspaces in an m-dimensional vector space V over $F_{q}$. Let $h_{i}\in U_{i}$ (i∈[r]), and suppose that $U_{0}$ is a subspace of $H:=\langle h_{1},\ldots ,h_{r}\rangle$ with $\dim U_{0}=\alpha$. Define $C\subseteq F_{q}^{r}$ to be the collection of all $c\in F_{q}^{r}$ for which $\sum_{i=1}^{r}c_{i}h_{i}\in U_{0}$. If every collection $\left\{U_{j}|j\in J\cup \left\{0\right\}\right\}$ with J⊆[r] and |J|=r−α is independent, then $h_{1},\ldots ,h_{r}$ are independent and C is an ${\left[r,\alpha ,r-\alpha +1\right]}_{q}$ MDS code.*

**Proof.**  Since $U_{0}$ is a subspace, the code *C* is linear over $F_{q}$. Suppose that (after renumbering if necessary) $h_{1},\ldots ,h_{t}$ form a basis of *H*, for some t≤r. Let $C_{0}$ be the subcode of *C* consisting of all c∈C with supp(c)⊆[t]}. Obviously, every $u\in U_{0}$ can be written as $u=\sum c_{i}h_{i}$ for a codeword $c\in C_{0}$, and since $h_{1},\ldots ,h_{t}$ are independent, every such expression is unique. As a consequence, $\dim C_{0}=\dim U_{0}=\alpha$. Moreover, if $C_{0}$ contains a nonzero codeword c with |supp(c)|≤r−α, then $U_{0}$ and the subspaces $U_{j}$ with j∈supp(c) are not independent, since the word $u\in U_{0}$ corresponding to the codeword c can be written as a linear combination of the vectors $h_{j}$ with j∈supp(c). Therefore, $C_{0}$ is a linear code of length at most *r*, of dimension α, and with minimum distance at least r−α+1. By the Singleton bound, we conclude that t=r and $C_{0}$ has minimum distance r−α+1. As a consequence, $h_{1},\ldots ,h_{r}$ are independent and $C_{0}=C$; hence *C* is an [r,α,r−α+1] MDS code over $F_{q}$.  □

**Remark** **3.** *We note that a similar result holds if β>1 and α=sβ. As before, we can describe $U_{0}$ in terms of an ${\left[r\beta ,s\beta \right]}_{q}$ code, with the positions partitioned into r groups of β positions each, but we can now only conclude that a nonzero codeword is nonzero in at least r−s+1 of these* groups*, and so the code need not be MDS. However, by considering the code an a code of length r over the larger symbol alphabet $F_{q^{\beta }}$, we see that the minimum symbol-weight of this $F_{q}$-linear but not $F_{q^{\beta }}$-linear code of length r and size ${\left(q^{\beta }\right)}^{s}$ is at least r−s+1, so the minimum symbol-distance is r−s+1. Therefore, this code meets the Singleton bound for non-linear codes [55] (Theorem 4.1), and is, again, a (non-linear) MDS code (or MDS array code). We leave further details to the interested reader.*

Lemma 9 has an interesting consequence.

**Corollary** **1.** *If there exists an optimal linear FR storage code with parameters ${\left\{m,\left(n,k\right),\left(r,\alpha ,1\right)\right\}}_{q}$ in a corner point of the achievable cut-set region (that is, with α integer), then there exists an ${\left[r,\alpha ,r-\alpha +1\right]}_{q}$ MDS code.*

**Proof.**  Suppose that $\pi =U_{1},\ldots ,U_{n-1}$ is a protostate of such a code. Then we can choose helpers $h_{i}\in U_{i}$ for i∈[r] and a subspace $U_{0}\subseteq H:=\langle h_{i}|i\in \left[r\right]\rangle$ with dimU=α such that $\sigma =U_{1},\ldots ,U_{0},\ldots ,U_{n-1}$ is a coding state of that code. By Lemma 1, any collection of subspaces $U_{j}$ (j∈J) with |J|=r−α+1 is independent. Now the desired conclusion follows from Lemma 9.  □

We are now ready to state our main result. This result was announced already in [48] (Theorem 4.1), but, unfortunately, the required extra condition on the helper nodes was inadvertently omitted.

**Theorem** **3.** *Suppose that $\pi =\{U_{1},\ldots ,U_{r}\}$ is (r,α)-regular in a vector space V of dimension $m=m_{r,\alpha }=\alpha \left(2r-\alpha +1\right)/2$ over a finite field $F_{q}$, and let $h_{i}\in U_{i}$ for i∈[r]. Define ${\bar{U}}_{i}$ as in (15). Then $U_{i}\backslash {\bar{U}}_{i}$ is nonempty for all i∈[r]. Let $C\subseteq F_{q}^{r}$ and let $U_{0}:=\left\{c_{1}h_{1}+\cdots +c_{r}h_{r}|c=\left(c_{1},\ldots ,c_{r}\right)\in C\right\}$. Then $\sigma :=\{U_{0},U_{1},\ldots ,U_{r}\}$ is an (r,α)-regular extension of π if and only if $h_{i}\in U_{i}\backslash {\bar{U}}_{i}$ for all i∈[r] and C is an [r,α,r−α+1] MDS code over $F_{q}$.*

**Proof.**  Note that by our assumption on π, we have $\dim{\bar{U}}_{i}=m-1$ and $\dim\langle {\bar{U}}_{i},U_{i}\rangle =\dim V=m$, hence $U_{i}$ is not contained in ${\bar{U}}_{i}$; so $U_{i}\backslash {\bar{U}}_{i}$ is nonempty.

We begin by showing that the conditions on the vectors $h_{i}$ (i∈[r]) and on *C* are necessary. So suppose that σ is (r,α)-regular. First, if $h_{i}\in {\bar{U}}_{i}$, then $U_{0}\subseteq {\bar{U}}_{i}$, hence $\sigma \backslash \{U_{i}\}$ is contained in the proper subspace ${\bar{U}}_{i}$ of *V*, so it is not an (r,α)-configuration, contradicting our assumption. Hence $h_{i}\in U_{i}\backslash {\bar{U}}_{i}$ for all *i*. Then by Lemma 4 with $H_{i}:=\langle h_{i}\rangle$ (i∈[r]), the vectors $h_{1},\ldots ,h_{r}$ are independent. Next, let $\bar{C}$ denote the collection of all $c\in F_{q}^{r}$ for which $\sum c_{i}h_{i}\in U_{0}$. Since $h_{1},\ldots ,h_{r}$ are independent, we have $\bar{C}=C$ and by Lemma 9, we have that $\bar{C}$, hence also *C*, is an ${\left[r,\alpha ,r-\alpha +1\right]}_{q}$ MDS code.

Now, we show that the conditions are also sufficient. So assume that $h_{i}\in U_{i}\backslash {\bar{U}}_{i}$ for all *i* and that *C* is [r,α,r−α+1] MDS. By Lemma 4 with $H_{i}=\langle h_{i}\rangle$ (i∈[r]), the vectors $h_{1},\ldots ,h_{r}$ are independent; hence $\dim U_{0}=\dim C=\alpha$. Next, let J⊆[r]∪{0} with |J|=r−α+1+t for some integer *t* with 0≤t≤k−r+s−1. According to Definition 5, we have to show that $\dim\langle U_{j}|j\in J\rangle =d_{t}=\left(r-\alpha +1\right)\alpha +\left(\alpha -1\right)+\cdots +\left(\alpha -t\right)$. If 0∉J, this holds since π is (r,α)-regular. So assume that $J=J_{0}\cup \left\{0\right\}$ with $J_{0}\subseteq \left[r\right]$ and $|J_{0}|=r-\alpha +t$. Again using that π is (r,α)-regular, we have $\dim\langle U_{j}|j\in J_{0}\rangle =d_{t-1}$, so by Grassmann’s identity,

(22) $$\dim\langle U_{j}|j\in J\rangle =d_{t-1}+\alpha -\dim\langle U_{j}|j\in J_{0}\rangle \cap U_{0},$$

which is also correct for t=0 if we set $d_{-1}:=\left(r-\alpha \right)\alpha$. Setting $H:=\langle h_{1},\ldots ,h_{r}\rangle$, we have $U_{0}\subseteq H$; hence, using Lemma 4 and setting $C_{0}:=\left\{c\in C|\mathrm{supp}\left(c\right)\subseteq J_{0}\right\}$, we have

(23) $$\langle U_{j}|j\in J_{0}\rangle \cap U_{0}=\langle U_{j}|j\in J_{0}\rangle \cap H\cap U_{0}=\langle h_{j}|j\in J_{0}\rangle \cap U_{0}=\left\{\sum c_{j}h_{j}|c\in C_{0}\right\}.$$

Now *C* is MDS and dimC=α; hence, $\dim C_{0}=\max(0,\alpha -(r-|J_{0}\left|)\right)=\max\left(0,t\right)=t$. So combining (22) and (23), we have

$$\dim U_{0}\cap \langle U_{j}|j\in J_{0}\rangle =d_{t-1}+\alpha -t=d_{t}.$$

Since $J_{0}$ is arbitrary, we conclude that σ is (r,α)-regular and of size r+1 as claimed.  □

This theorem has the following important consequence.

**Theorem** **4.** *Let $F_{q}$ be the finite field of size q. Suppose that there exists an [r,α,r−α+1] MDS code C over $F_{q}$. Then the family of all (r,α)-configurations of size r+1 in a vector space V of dimension $m=m_{r,\alpha }=\left(r-\alpha +1\right)\alpha +\left(\alpha -1\right)+\cdots +\left(r-k+1\right)$ over $F_{q}$ forms the collection of coding states of an optimal linear storage code over $F_{q}$ with parameters ${\left\{m,\left(r+1,r\right),\left(r,\alpha ,1\right)\right\}}_{q}$. The protostates of his code are the (r,α)-regular configurations of size r.*

**Proof.**  In Theorem 1, we showed how to use an ${\left[r,\alpha ,r-\alpha +1\right]}_{q}$ MDS code *C* to construct an (r,α)-regular configuration of size r+1, so the collection of coding states in the theorem is nonempty. And if a coding space is lost, then we are left with a protostate, which is (r,α)-regular of length *r*, and we can use Theorem 3 and the MDS code *C* to repair this protostate to another coding state.  □

It is usually possible to use a *subset* of the collection of all (r,α)-configurations of length r+1 as coding states. A rather obvious restriction is discussed in the remark below.

**Remark** **4.** *In Theorem 4, we can limit the coding states to all (r,α)-regular collections of size r+1 in V that can be obtained by repair from a subcollection of size r, since other ones are not reachable. For example, let $V=F_{2}^{5}$, and let $a_{1},a_{2},e_{1},e_{2},e_{3}$ be a basis for V; set $a_{3}:=a_{1}+a_{2}$. For i∈[3], define $U_{i}:=\langle a_{i},e_{i}\rangle$, define $U_{4}:=\langle e_{1}+e_{2},a_{1}+e_{1}+e_{3}\rangle$, and define $U_{4}^{\prime }:=\langle e_{1}+e_{2},e_{1}+e_{3}\rangle$. It is easily verified that both $\pi :=\{U_{1},U_{2},U_{3},U_{4}\}$ and $\pi^{\prime }:=\left\{U_{1},U_{2},U_{3},U_{4}^{\prime }\right\}$ are (3,2)-regular of size 4 (in fact, it can be shown that, up to a linear transformation,* every* (3,2)-regular configuration is equal to either π or $\pi^{\prime }$), and, moreover, no subspace $U_{i}$ (i∈[4]) can be obtained by 1-repair from the other three subspaces in π. So there is no need to include configurations such as π as coding states of a ${\left\{5,\left(4,3\right),\left(3,2,1\right)\right\}}_{2}$ storage code.*

In view of Theorem 3, Theorem 4, and of Remark 4, we introduce the following.

**Definition** **8.** *Let r and α be integers with 1≤α≤r. An optimal linear storage code with parameters $\left\{m_{r,\alpha },\left(r+1,r\right),\left(r,\alpha ,1\right)\right\}$ is called an* (r,α)-regular storage code *if the code has an ambient space V with $\dim V=m_{r,\alpha }$ and if every coding state is an (r,α)-regular configuration in V.*

In the next section, we will introduce a more interesting family of (r,α)-regular storage codes.

We end this section with two further remarks.

**Remark** **5.** *We show in Theorem 3 that an (r,α)-regular storage code over a finite field $F_{q}$ exists if and only if an ${\left[r,\alpha ,r-\alpha +1\right]}_{q}$ MDS code exists. As rightly pointed out by a reviewer, that leaves open the possibility that a storage code with parameters ${\left\{m_{r,\alpha },\left(r+1,r\right),\left(r,\alpha ,1\right)\right\}}_{q}$ exists while no ${\left[r,\alpha ,r-\alpha +1\right]}_{q}$ MDS code exists. We are not aware of any non-existence results for regenerating codes in terms of the alphabet size (even for MBR codes, this is listed as Open Problem 1 in [29]), so we cannot rule out this possibility. If one could prove that (9) always holds with equality, then we could conclude that every* linear *$\left\{m_{r,\alpha },\left(r+1,r\right),\left(r,\alpha ,1\right)\right\}$ storage code is (r,α)-regular, but we do not see how to prove that (if it is true at all, which we doubt). But given the strong relation between construction methods for storage codes and MDS codes, and given our idea that these (r,α)-regular codes are, in a sense, “best-possible”, we strongly believe that these codes indeed realize the smallest possible alphabet size for their parameters. We leave this question as an interesting open problem.*

**Remark** **6.** *Interestingly, every storage code as in Theorem 3 can be realized as an* optimal-access *code, and, in fact, as a* help-by-transfer *(HBT) code. Essentially, with notation as in Theorem 3, the reason is that if a coding space $U_{i}$ is represented by a basis $e_{1},\ldots ,e_{\alpha }$, then since $U_{i}⊊{\bar{U}}_{i}$, there must be an index j∈[α] such that $e_{j}\in U_{i}\backslash {\bar{U}}_{i}$. Note that this property need not hold for* every *(r,α)-regular storage code, since it may be required to choose helper vectors outside the given basis in order to repair to an* available *coding state. An example of this is given by the (r,α)=(3,2)-regular code from [48], as can be seen from its description in [50]. It is an interesting problem to find the* smallest *(3,2)-regular HBT code. We leave further details to the interested reader.*

## 8. Smaller (r,α)-Regular Storage Codes

Inspired by Theorem 2, we will use Theorem 3 to produce a second (essentially recursive) construction of an (r,α)-regular collection of size r+1.

To this end, let *V* be a vector space over $F_{q}$ with $\dim V=m_{r,\alpha }$. For t=1,…,α, let $C^{\left(\delta +t\right)}$ be an ${\left[\delta -1+t,t,\delta \right]}_{q}$ MDS code, where δ=r−α+1. In what follows, we will consider bases *H* for *V* consisting of vectors $h_{i,j}$ for i=1,…,α and j=1,…,δ−1+i, arranged as in Table 1.

Recall that by Lemma 3, we have $m_{r,\alpha }=\delta +\left(\delta +1\right)+\cdots +r$, so by counting “by row”, we see that these bases indeed have the right size. Given such a basis $H=(h_{i,j})$, we can use the given MDS codes to construct a sequence $\sigma =\sigma \left(H,C^{\left(\delta +1\right)},\ldots ,C^{\left(r+1\right)}\right)=U_{1},\ldots ,U_{r+1}$ as follows. First, for t=1,…,δ, we let

(24) $$U_{t}:=\langle h_{i,t}|i\in \left[\alpha \right]\rangle .$$

Then, for t=1,…,α, we define

(25) $$W_{\delta +t}:=\left\{\sum_{j=1}^{\delta -1+t}c_{j}h_{t,j}|c=\left(c_{1},\ldots ,c_{\delta -1+t}\right)\in C^{\left(\delta +t\right)}\right\}$$

and we let

(26) $$U_{\delta +t}:=\langle W_{\delta +t},h_{t+1,\delta +t}\ldots ,h_{\alpha ,\delta +t}\rangle .$$

**Lemma** **10.** *With the above notation and assumptions, we have $\dim W_{\delta +t}=\dim C^{\left(\delta +t\right)}=t$ (t∈[α]), and the collection $\sigma :=\{U_{1},\ldots ,U_{r+1}\}$ is (r,α)-regular.*

**Proof.**  First, since $h_{t,1},\ldots ,h_{t,\delta -1+t}$ are independent, it follows that $\dim W_{\delta +t}=\dim C^{\left(\delta +t\right)}$; hence $\dim W_{\delta +t}=C^{\left(\delta +t\right)}=t$. Then, from (24), we see that $\dim U_{t}=\alpha$ for t∈[α], and from (26), we see that $\dim U_{\delta +t}=t+\left(\alpha -\left(t+1\right)+1\right)=\alpha$, so all the subspaces in σ have the required dimension α. We will use induction to prove the last claim. To establish the base case for the induction, note that the δ+1 subspaces $U^{\left(1\right)}:=\langle h_{1,1}\rangle ,\ldots ,U^{\left(\delta \right)}:=\langle h_{1,\delta }\rangle ,U^{\left(\delta +1\right)}:=W_{\delta +1}$ form a (δ,1)-regular configuration (indeed, since $C^{\left(\delta +1\right)}$ is MDS with dimension 1, the unique (up to a scalar) nonzero codeword in $C^{\left(\delta +1\right)}$ has weight δ, hence is nonzero in every position). Now, suppose that we have constructed a (δ−1+t,t−1)-regular configuration $\sigma^{\left(t\right)}:=\left\{U_{1}^{\left(t-1\right)},\ldots ,U_{\delta -1+t}^{\left(t-1\right)}\right\}$. Then, we “add an extra layer” by setting $U_{j}^{\left(t\right)}:=\langle U_{j}^{\left(t-1\right)},h_{t,j}\rangle$ (j∈[δ−1+t]), we add an extra subspace $U_{\delta +t}^{\left(t\right)}:=W_{\delta +t}$, and we apply Theorem 2, part (i) to conclude that $\sigma^{\left(t\right)}:=\left\{U_{1}^{\left(t\right)},\ldots ,U_{\delta +t}^{\left(t\right)}\right\}$ is (δ+t,t)-regular. Since $\sigma^{\left(\alpha \right)}=\sigma$, the claim follows by induction.  □

Next, we want to show that by restricting the allowed MDS codes involved, we can construct an (r,α)-regular storage code using only coding states of the type in Lemma 10. In that case, a coding state of this restricted type, when losing a subspace, must be repairable to a new coding state that is again of this restricted type. We will now sketch how this can be achieved.

Let *C* be a fixed [r,α,δ] MDS code *C*. For every permutation $\tau =\tau_{1},\ldots ,\tau_{r}$ of {1,…,r}, we define codes $C^{\left(\delta +1\right)},\ldots ,C^{\left(r+1\right)}$ by letting

(27) $$C^{\left(\delta +t\right)}:=\left\{\left(c_{\tau_{1}},\ldots ,c_{\tau_{\delta -1+t}}\right)|c=\left(c_{1},\ldots ,c_{r}\right)\in C,\mathrm{supp}\left(c\right)\subseteq \left\{\tau_{1},\ldots ,\tau_{\delta -1+t}\right\}\right\}.$$

Note that since *C* is MDS, the code $C^{\left(\delta +t\right)}$ is easily seen to be [δ−1+t,t,δ] MDS; note also that $C^{\left(r+1\right)}=C$. Now, for every basis $H=\{h_{i,j}|1\leq i\leq \alpha ,1\leq j\leq \delta -1+i\}$ for *V*, we use these codes $C^{\left(\delta +t\right)}$ defined above to construct an (r,α)-regular configuration σ=σ(H,τ) as explained earlier, that is, we set $\sigma \left(H,\tau \right):=\sigma \left(H,C^{\left(\delta +1\right)},\ldots ,C^{\left(r+1\right)}\right)$. Then by Lemma 10, σ(H,τ) is (r,α)-regular. We now have the following.

**Theorem** **5.** *Let r and α be integers with 1≤α≤r, let V be a vector space over $F_{q}$, with $\dim V=m_{r,s}$, and let C be an ${\left[r,\alpha ,\delta \right]}_{q}$ MDS code, so with δ=r−α+1. The collection of all (r,α)-regular configurations of the form σ(H,τ) as defined above, where $H=(h_{i,j}|i\in \left[\alpha \right],j\in \left[\delta -1+i\right])$ is a basis for V and where τ is a permutation of [r], forms an (r,α)-regular storage code.*

**Proof.**  We sketch a proof as follows. Suppose that for each t∈[α], we choose a basis $s_{1,\delta +t},\ldots ,s_{t,\delta +t}$ for $W_{\delta +t}$. Then

$$U_{\delta +t}=\langle s_{1,\delta +t},\ldots ,s_{t,\delta +t},h_{t+1,\delta +t}\ldots ,h_{\alpha ,\delta +t}\rangle .$$

Note that every vector $s_{u,\delta +t}$ can be uniquely expressed as a linear combination of the basis vectors $h_{i,j}$ for *V*; we will say that a vector $h_{i,j}$*occurs in $s_{u,\delta +t}$* if $h_{i,j}$ occurs in that linear combination with a *nonzero* coefficient. Later, we will impose additional conditions on these vectors $s_{u,\delta +t}$.

We can now arrange the vectors $h_{i,j}$ and the vectors $s_{i,\delta +j}$ in a rectangular α×(r+1) array such that the vectors in column *j* span $U_{j}$, see Table 2 below.

This array has the following characteristics.

A1 Row *i* of the array contains δ−1+i of the basis vectors of *V*.
A2 The basis vectors in row *i* occur only in the vectors $s_{1,\delta +i},\ldots ,s_{i,\delta +i}$.
A3 The vector space $W_{\delta +i}=\langle s_{1,\delta +i},\ldots ,s_{i,\delta +i}\rangle$ is determined by the basis vectors in row *i* and by an [δ−1+i,i,δ] MDS code $C^{\left(\delta +i\right)}$ derived from the [r,α,δ] MDS code *C* through a fixed permutation τ of {1,…,r}.

Now consider what happens if we lose a subspace, that is, if we lose a column of the array in Table 2. Our aim will be to arrange the remaining *r* subspaces into a similar array, but with the last column removed, and then to use the MDS code *C* to construct the last column from the last row of the new array. Losing any column *j* with j≤r has the consequence of losing the basis vectors $h_{i,j}$ in the array, and our aim will be to replace these lost basis vectors with the vectors $s_{u,\delta +t}$ (where u=1 if j≤δ and u=j−δ if δ+1≤j≤r), while maintaining the characteristics A1–A3 above. By A1, a row that contains a lost variable should move one row up, and the row that contains the replacement basis vectors should move into the last row. By A2, if $s_{u,\delta +t}$ replaces $h_{i,j}$, then $h_{i,j}$ should occur in $s_{u,\delta +t}$ and should not occur in $s_{s,\delta +t}$ for s≠u. Note that since $C^{\left(\delta +i\right)}$ is an MDS code, there is no position where all codewords have a 0; hence we can always choose a basis $s_{1,\delta +i},\ldots ,s_{i,\delta +i}$ for $W_{\delta +i}$ such that a given vector $h_{i,j}$ occurs in one and in only one of the basis vectors. Finally, by A3, there has to be a suitable permutation $\tau^{\prime }$ that can describe the new [δ−1+t,t,δ] MDS codes. As we saw above, A1 and A2 determine how the new array should be formed; what is left is to find a suitable $\tau^{\prime }$, and then to verify that A3 holds again. Let us now turn to the details.

As remarked before, if we lose $U_{r+1}$, then we can recover that subspace *exactly*. For the other subspaces, we distinguish two cases.

First, suppose we lose a subspace $U_{t}$ with 1≤t≤δ. Then, in Table 2, we delete column *t*, and we take out row 1 and place it after the last row, where we want the α vectors $s_{1,\delta +1},\ldots ,s_{1,r+1}$ to replace the lost basis vectors $h_{1,t},\ldots ,h_{\alpha ,t}$. Recall that the vectors $s_{1,\delta +i},\ldots ,s_{i,\delta +i}$ span $W_{\delta +i}$ and are each a linear combination of $h_{i,1},\ldots ,h_{i,\delta -1+u}$; now, choose these vectors such that $s_{u,\delta +i}$ contains $h_{i,t}$ if and only if u=1 (as remarked above, it is not difficult to verify that this is possible). Define a new permutation

(28) $$\tau^{\prime }=\tau_{1},\ldots ,\tau_{t-1},\tau_{t+1},\ldots ,\tau_{r},\tau_{t},$$

and a new basis $H^{\prime }=\left(h_{i,j}\right)$, where, for i=1,…,α−1, j=1,…,δ−1+i, we let

$$h_{i,j}^{\prime }=\left\{\begin{array}{cc} h_{i+1,j}, & \mathrm{if}\;j<t;\\ h_{i+1,j+1}, & \mathrm{if}\;j>t \end{array}\right.$$

and for j=1,…,r, we let

$$h_{\alpha ,j}^{\prime }=\left\{\begin{array}{cc} h_{1,j}, & \mathrm{if}\;j<t;\\ h_{1,j+1}, & \mathrm{if}\;t<j<\delta ;\\ s_{1,j+1}, & \mathrm{if}\;j\geq \delta . \end{array}\right.$$

Finally, with

(29) $$U_{0}:=\left\{\sum_{s=1}^{r}c_{\tau_{s}}h_{\alpha ,s}^{\prime }|c\in C\right\},$$

it is easily verified that $\sigma^{\prime }:=U_{1},\ldots ,U_{t-1},U_{t+1},\ldots ,U_{r+1},U_{0}$ is precisely the configuration $\sigma (\tau^{\prime },H^{\prime })$.

Secondly, suppose that we lose subspace $U_{\delta +t}$ with 1≤t≤α. In that case, we proceed in a similar way, where in Table 2 we remove column δ+t, take out row *t* and place that row after the last row in the table, where we now want the α−t vectors $s_{t,\delta +t+1},\ldots ,s_{t,r+1}$ to replace the lost basis vectors $h_{t+1,\delta +t},\ldots ,h_{\alpha ,\delta +t}$. This can be achieved by now choosing $s_{u,\delta +i}$ to contain $h_{i,\delta +t}$ if and only if u=t. Define a new permutation

(30) $$\tau^{\prime }=\tau_{1},\ldots ,\tau_{\delta +t-1},\tau_{\delta +t+1},\ldots ,\tau_{r},\tau_{\delta +t},$$

and a new basis $H^{\prime }=\left(h_{i,j}\right)$, where for i=1,…,α−1, j=1,…,δ−1+i, we let

$$h_{i,j}^{\prime }=\left\{\begin{array}{cc} h_{i,j}, & \mathrm{if}\;i<t,j<\delta +t;\\ h_{i,j+1}, & \mathrm{if}\;i<t,j>\delta +t;\\ h_{i+1,j+1}, & \mathrm{if}\;i>t,j>\delta +t \end{array}\right.$$

and

$$h_{\alpha ,j}^{\prime }=\left\{\begin{array}{cc} h_{t,j}, & \mathrm{if}\;j<\delta +t;\\ s_{t,j+1}, & \mathrm{if}\;\delta +t<j<r. \end{array}\right.$$

With $U_{0}$ as in (29), it is again easily verified that $\sigma^{\prime }:=U_{1},\ldots ,U_{\delta +t-1},U_{\delta +t+1},\ldots ,U_{r+1}$, $U_{0}$ is precisely the configuration $\sigma (\tau^{\prime },H^{\prime })$.

We leave further details to the reader.  □

It turns out that with a proper choice for the MDS code *C*, the (r,α)-regular configurations described in Theorem 5 may possess extra symmetry, even to the point where they are all equal up to a linear transformation, for example, when q=2, r−α=1, and the MDS code *C* is the *even weight* ${\left[r,r-1,2\right]}_{2}$ MDS code. In such cases, we can apply automorphism group techniques to construct “small” (r,α)-regular storage codes that involve only a relatively small number of different coding spaces. Examples of storage codes constructed in this way are the small (3,2)-regular code from [48] that involves only 8 different coding spaces, and the small (4,3)-regular storage code from [49,50] that involves only 72 different coding spaces. For more details on how such codes can be constructed, using groups of linear transformations fixing a protostate, we refer to [48,49,50].

## 9. Conclusions

A regenerating storage code (RGC) with parameters ${\left\{m,\left(n,k\right),\left(r,\alpha ,\beta \right)\right\}}_{q}$ is designed to store *m* data symbols from a finite field $F_{q}$ in encoded form on *n* storage nodes, each storing α encoded symbols. If a node is lost, a replacement node may be constructed by obtaining β symbols from each of a collection of *r* of the surviving nodes, called the *helper nodes*. The name of these codes stems from the requirement that, even after an arbitrary amount of repairs, any *k* nodes can regenerate the original data. We say that the code employs *exact repair (ER)* if, after each repair, the information on the replacement node is *identical* to the information on the lost node; if not, then we say that the code employs *functional repair (FR)*. An RGC is called *optimal* if its parameters meet an upper bound called the *cut-set bound*.

Linear MDS codes have often been instrumental in the construction of optimal RGC’s. In this paper, we first introduce a special type of configurations of vector spaces that we call *(r,α)-regular*. We show that such configurations can be constructed from suitable linear MDS codes. Then we employ linear MDS codes and (r,α)-regular configurations to construct what we call *(r,α)-regular codes*, which are optimal linear RGC’s with n−1=k=r and β=1, over a relatively small finite field $F_{q}$ (if r−α≤1, then any field can be used; if r−α>1, then q≥r−1 is required). Along the way, we show that, conversely, the existence of an (r,α)-regular code over a finite field of size *q* implies the existence of an ${\left[r,\alpha ,r-\alpha +1\right]}_{q}$ MDS code over that field.

Apart from two known examples, these storage codes are the only known explicit optimal RGC’s with parameters realizing an extremal point of the achievable cut-set region different from the MSR and MBR points.

## Figures and Tables

**Table 1:** The array of basis vectors.

$h_{1,1}$	⋯	$h_{1,\delta }$				
⋮		⋮	⋱			
$h_{t,1}$	⋯	$h_{t,\delta }$	⋯	$h_{t,\delta -1+t}$		
⋮		⋮			⋱	
$h_{\alpha ,1}$	⋯	$h_{\alpha ,\delta }$	⋯	$h_{\alpha ,\delta -1+t}$	⋯	$h_{\alpha ,r}$

**Table 2:** The array of vectors constructed above.

$U_{1}$		$U_{\delta }$	$U_{\delta +1}$		$U_{\delta -1+t}$	$U_{\delta +t}$	$U_{\delta +t+1}$		$U_{r+1}$
$h_{1,1}$	⋯	$h_{1,\delta }$	$s_{1,\delta +1}$	⋯	$s_{1,\delta -1+t}$	$s_{1,\delta +t}$	$s_{1,\delta +t+1}$	⋯	$s_{1,r+1}$
⋮		⋮	⋱		⋮	⋮	⋮		⋮
$h_{t,1}$	⋯	$h_{t,\delta }$		⋯	$h_{t,\delta -1+t}$	$s_{t,\delta +t}$	$s_{t,\delta +t+1}$	⋯	$s_{t,r+1}$
$h_{t+1,1}$	⋯	$h_{t+1,\delta }$		⋯	$h_{t+1,\delta -1+t}$	$h_{t+1,\delta +t}$	$s_{t+1,\delta +t+1}$	⋯	$s_{t+1,r+1}$
⋮		⋮			⋮	⋮	⋮		⋮
$h_{\alpha ,1}$	⋯	$h_{\alpha ,\delta }$	$h_{\alpha ,\delta +1}$	⋯	$h_{\alpha ,\delta -1+t}$	$h_{\alpha ,\delta +t}$	$h_{\alpha ,\delta +t+1}$	⋯	$s_{\alpha ,r+1}$

## Data Availability

No new data were created or analyzed in this study. Data sharing is not applicable to this article.

## Abbreviations

The following abbreviations are used in this manuscript:

MDPI	Multidisciplinary Digital Publishing Institute
DOAJ	Directory of open access journals
TLA	Three letter acronym
LD	Linear dichroism
